# Saliva is more sensitive than nasopharyngeal or nasal swabs for diagnosis of asymptomatic and mild COVID-19 infection

**DOI:** 10.1038/s41598-021-82787-z

**Published:** 2021-02-04

**Authors:** Alvin Kuo Jing Teo, Yukti Choudhury, Iain Beehuat Tan, Chae Yin Cher, Shi Hao Chew, Zi Yi Wan, Lionel Tim Ee Cheng, Lynette Lin Ean Oon, Min Han Tan, Kian Sing Chan, Li Yang Hsu

**Affiliations:** 1 Saw Swee Hock School of Public Health, National University of Singapore, National University Health System, #10-01, 12 Science Drive 2, Singapore, 117549 Singapore; 2 Lucence Diagnostics, Singapore, Singapore; 3 Department of Medical Oncology, National Cancer Centre, Singapore, Singapore; 4 Duke-NUS Graduate Medical School, National University of Singapore, Singapore, Singapore; 5 Genome Institute of Singapore, Singapore, Singapore; 6 Headquarters Army Medical Services, Singapore Armed Forces, Singapore, Singapore; 7 Department of Diagnostic Radiology, Singapore General Hospital, Singapore, Singapore; 8 Department of Molecular Pathology, Singapore General Hospital, Singapore, Singapore; 9 Yong Loo Lin School of Medicine, National University of Singapore, National University Health System, Singapore, Singapore

**Keywords:** Diagnosis, Viral infection

## Abstract

We aimed to test the sensitivity of naso-oropharyngeal saliva and self-administered nasal (SN) swab compared to nasopharyngeal (NP) swab for COVID-19 testing in a large cohort of migrant workers in Singapore. We also tested the utility of next-generation sequencing (NGS) for diagnosis of COVID-19. Saliva, NP and SN swabs were collected from subjects who presented with acute respiratory infection, their asymptomatic roommates, and prior confirmed cases who were undergoing isolation at a community care facility in June 2020. All samples were tested using RT-PCR. SARS-CoV-2 amplicon-based NGS with phylogenetic analysis was done for 30 samples. We recruited 200 subjects, of which 91 and 46 were tested twice and thrice respectively. In total, 62.0%, 44.5%, and 37.7% of saliva, NP and SN samples were positive. Cycle threshold (Ct) values were lower during the earlier period of infection across all sample types. The percentage of test-positive saliva was higher than NP and SN swabs. We found a strong correlation between viral genome coverage by NGS and Ct values for SARS-CoV-2. Phylogenetic analyses revealed Clade O and lineage B.6 known to be circulating in Singapore. We found saliva to be a sensitive and viable sample for COVID-19 diagnosis.

## Introduction

The severe acute respiratory syndrome coronavirus 2 (SARS-CoV-2) emerged from Wuhan, China, in November 2019^1^, and has since caused a global pandemic, with over 25 million confirmed COVID-19 cases and 850,000 deaths as of 1st September 2020^2^. Singapore has since recorded over 56,000 cases and 27 deaths since the first case was reported on 23rd January 2020, the majority of cases being migrant workers living in crowded dormitories^3^.

Acute coronavirus disease 2019 (COVID-19) is primarily diagnosed via reverse transcription-polymerase chain reaction (RT-PCR) detection of viral genetic material. However, considering the three primary modes of transmission of SARS-Cov-2 i.e., contact, droplet and aerosol routes, various types of samples have been suggested for the purpose of detection^4^. In Singapore and several other countries, nasopharyngeal (NP) swabs are the principal means for collecting specimens for testing^5,6^. However, the collection procedure for NP swabs can cause discomfort and require trained healthcare staff to perform.

Saliva and self-administered nasal (SN) swabs are, in many ways, ideal specimens for COVID-19 screening. Both can be collected safely without the need for trained staff. The utility of saliva for COVID-19 testing has been tested in multiple territories and countries^7–15^. The majority of current published studies involve relatively small numbers of subjects. A meta-analysis suggests that saliva is at best slightly less sensitive or similar to other specimens, including NP swabs^16^. However, one caveat relates to how saliva is collected—saliva is a complex bio-mixture which can consist of salivary gland secretion, gingival crevicular fluid, sputum and/or mucosal transudate, in varying proportions depending on collection method. Some studies tested only secretions from the mouth^11,12^, others explicitly tested “posterior oropharyngeal” or “deep throat” saliva with secretions from the oropharynx^7–10^, while the rest were unspecified^13–16^.

We aimed to test the sensitivity of “naso-oropharyngeal” saliva and SN swabs compared to NP swabs in a large cohort of migrant workers in Singapore using RT-PCR testing. We additionally used direct-from-RNA amplicon-based next-generation sequencing (NGS) for confirmatory detection of low-level SARS-CoV-2 signal and to establish phylogeny for tested samples.

## Methods

### Study population

Subjects were recruited between 2nd and 26th June 2020 from two sites—a 5400-bed purpose-built dormitory where migrant workers were housed in large rooms holding 7–20 workers, and a community care facility (CCF) where migrant workers diagnosed with COVID-19 but not requiring acute hospital care were sent for isolation and monitoring. All subjects at the CCF are prior confirmed cases (via RT-PCR), while subjects from the dormitory comprised two groups—(1) migrant workers presenting with symptoms of acute respiratory tract infection (ARI); and (2) asymptomatic roommates of newly diagnosed COVID-19 cases.

### Ethics statement

This study was approved by the Director of Medical Services, Ministry of Health, under Singapore’s Infectious Disease Act^17^. Under this Act, in the event of a major outbreak, the Director may require the obtainment of such information or samples (including human samples) as deemed appropriate or necessary that will be of significant public health benefit to the country^17^. Informed consent was obtained from all participants, and all methods were performed in accordance to Singapore guidelines and regulations for biomedical research.

### Sample collection

Migrant workers from the purpose-built dormitory presenting with ARI were assessed by physicians at the medical post, who made the decision for whether diagnostic NP swabs for COVID-19 testing was necessary. Those workers requiring NP swabs were immediately approached for study participation, and consent was taken where agreeable.

For the collection of SN swabs, participants were instructed to insert the swab (about 1 cm) into their nostrils (one at a time), tilt their head back slightly, and rotate the swab in a circular motion for 3 times around the nasal wall. The swab was then inserted into the collection tube. For the collection of naso-oropharyngeal saliva samples, participants were asked to tilt their head back slightly, clear their throat and nose, and spit the saliva into the collection bottle. The steps were repeated until the required volume (2 mL) was achieved. For “naso-oropharyngeal” saliva collection, instructional videos (video link in English: https://youtu.be/4jGrJUbjBBs) in the major native languages of the migrant workers were shown, following which these samples were collected under the supervision of a trained researcher.

For consenting subjects from CCF and asymptomatic roommates of newly diagnosed cases at the dormitory, NP swab collection procedure was performed by a trained researcher. SN swab and saliva samples were collected in the same sitting.

Each subject was tested up to three times at 2–3-days interval where possible, in order to compare the sensitivity of different samples across time. Subjects from the purpose-built dormitory who tested negative across all three samples during the first round of testing were not retested. Subjects from the CCF were not retested if all samples from the initial two rounds were negative.

NP swabs from subjects with ARI were sent dry in cooler boxes to the Singapore General Hospital (SGH) molecular laboratory as part of routine clinical testing. NP swabs and self-administered nasal swabs from other subjects were sent in 3 mL of viral transport medium, while up to 2 mL of saliva was collected in a container with 2 mL of viral RNA stabilization fluid (SAFER-Sample Stabilization Fluid, Lucence, Singapore) before transfer to Lucence. All samples were processed within the same day. Both service laboratories are the College of American Pathologists (CAP) accredited, and Lucence is CLIA-licensed.

### Laboratory testing

RT-PCR at SGH was performed using the automated cobas 6800 system (Roche, Branchburg, NJ, USA) on an automated cobas 6800 system, with results inferred according to the manufacturer’s specifications. NP and saliva samples sent to Lucence Laboratory underwent RNA extraction (200 μL of the sample) (GeneAid Biotech Ltd) and were tested with a laboratory-developed RT-PCR test (CDC-LDT) based on primers published by the Division of Viral Diseases, National Center for Immunization and Respiratory Diseases, Centers for Disease Control and Prevention, Atlanta, GA, USA^18^, while saliva and SN swabs were additionally tested using the Fortitude 2.1 kit (MiRXES, Singapore). The analytical limit of detection of the CDC-LDT was determined to be 25 copies per reaction based on a synthetic SARS-CoV-2 genome (Twist Bioscience). Saliva was pre-processed with the addition of dithiothreitol (DTT) at 0.4–0.85% of total sample volume, vortexing, and incubation at room temperature for 15 min. Solubilization was visibly apparent post-treatment at room temperature and RNA was extracted immediately post-treatment.

A limited number of samples was selected for the initial stage of determining performance specifications for a NGS-based SARS-CoV-2 assay. Both saliva and SN swab samples were included to demonstrate compatibility of RNA extracts from samples collected in the viral RNA stabilization fluid. Thirty samples were selected including high and low viral load samples, and those that had discordant results from the two RT-PCR assays. Six of the 30 samples were paired sets of saliva and SN swab samples from the same time point for 3 individuals, and 6 samples (4 saliva, 2 SN swabs) were collected at different time points from 3 other individuals. The remaining 18 samples comprised 10 saliva, 7 SN swab, and 1 NP swab sample from individual patients.

SARS-CoV-2 amplicon-based NGS was done using 330 primer pairs to generate amplicons (size range 130–178 bp) covering the entire virus genome (except the first 25 bases and 30 bases upstream of the final polyA tail) to establish a direct-from-sample workflow. To rule out potential non-specific amplification of other viruses related in sequence, all amplicons were verified to have limited similarity to sarbecoviruses, outside of SARS-related coronaviruses (assumed not to be present in circulation). The threshold coverage (%) for making positive call by NGS was established by performing NGS on 5 negative (by RT-PCR) samples from this study, 11 negative NP swab samples from community testing, and 10 no-template controls (NTC). For samples with complete viral genomes (100% coverage ≥ 1 × coverage), phylogenetic analysis was performed to identify lineages based on sequence variants.

### Statistical methods

We described our data using frequencies/percentages and median/interquartile range. We assessed the comparability between sampling methods using kappa-statistic and percent agreement. STATA 13.1 (StataCorp, Texas, USA) was used for all statistical calculations.

## Results

We recruited 200 subjects—149 from the dormitory and 51 from CCF. There were 45 subjects with ARI and 104 asymptomatic close contacts recruited from the purpose-built dormitory, while 51 subjects with confirmed COVID-19 (8 asymptomatic at the time of diagnosis) were recruited at the CCF (Table 1).

**Table 1:** Characteristics of recruited subjects.

Characteristics	Purpose-built dormitory (*n* = 149)	CCF (*n* = 51)
Male sex (%)	149 (100)	51 (100)
Median age (IQR), years	32 (28–37)	38 (36–41)
Symptomatic (%)	45 (30.2)	43 (84.3)
**Number of subjects tested**		
Round 1	149	51
Round 2	40	51
Round 3	1	45
**Number of symptomatic subjects with any positive test (%)**		
Round 1	30/74 (40.5)	37/42 (88.1)
Round 2	15/32 (46.9)	37/41 (90.2)
Round 3	0 (0)	37/41 (90.2)
**Number of asymptomatic subjects with any positive test (%)**		
Round 1	44/74 (59.5)	5/42 (11.9)
Round 2	17/32 (53.1)	4/41 (9.8)
Round 3	1/1 (100)	4/41 (9.8)
**Median (IQR) duration from symptom onset to test date, days—symptomatic subjects**		
Round 1	3 (2–4)	7 (6–8)
Round 2	5 (4–6)	10 (9–11)
Round 3	–	13 (12–14)
**Median (IQR) duration from date of initial diagnosis**^**a**^** to test date, days—asymptomatic subjects**		
Round 1	0 (0–0)	5.5 (5–6)
Round 2	4 (3–6)	8.5 (8–9)
Round 3	11 (11)	11.5 (11–12)

*CCF* community care facility, *IQR* interquartile range.

^a^For asymptomatic subjects recruited at the purpose-built dormitory, all three samples (nasopharyngeal, self-administered nasal, and saliva) were taken at the same time during round 1. For asymptomatic subjects recruited at CCF, subjects were diagnosed with COVID-19 via prior RT-PCR testing and only two samples (self-administered nasal and saliva) were taken during round 1.

Of 200 subjects, 91 and 46 completed second and third rounds of testing, respectively, resulting in 337 sets of tests (Table 2). Because COVID-19-positive migrant workers were rapidly transferred out of dormitories to CCFs, all but one subject from the dormitory site did not complete the planned testing. The median time period between the date of diagnosis and the first round of testing in asymptomatic subjects was 0 days (range 0–6 days), while the median time period between symptom onset and the first round of testing was 5.5 days (range 0–28 days).

**Table 2:** Results of RT-PCR testing on samples.

	Tests on symptomatic individuals (*n* = 188)	Tests on asymptomatic individuals (*n* = 149)
**Positive results, n (%)**		
Saliva—CDC-LDT	145 (77.1)	64 (43.0)
Saliva—Fortitude 2.1	123 (65.4)	44 (29.5)
NP swab—CDC-LDT	123 (65.4)	27 (18.1)
SN swab—CDC-LDT	96 (51.1)	31 (20.8)
SN swab—Fortitude 2.1	98 (52.1)	21 (14.1)
**Concordance of results among NP swabs (CDC-LDT), SN swabs (CDC-LDT), and saliva (CDC-LDT), n (%)**		
Saliva only positive	24 (12.8)	35 (23.5)
NP swab only positive	6 (3.2)	1 (0.7)
SN swab only positive	2 (1.1)	8 (5.4)
Saliva and NP swab positive	29 (15.4)	8 (5.4)
NP and SN swab positive	2 (1.1)	2 (1.3)
Saliva and SN swab positive	6 (3.2)	5 (3.4)
All three swabs positive in-tandem	86 (45.7)	16 (10.7)
**Concordance of results among NP swabs (CDC-LDT), SN swabs (Fortitude 2.1), and saliva (CDC-LDT), n (%)**		
Saliva only positive	25 (13.3)	39 (26.2)
NP swab only positive	5 (2.7)	1 (0.7)
SN swab only positive	1 (0.5)	4 (2.7)
Saliva and NP swab positive	26 (13.8)	10 (6.7)
NP and SN swab positive	3 (1.6)	2 (1.3)
Saliva and SN swab positive	5 (2.7)	1 (0.7)
All three swabs positive in-tandem	89 (47.3)	14 (9.4)
**Concordance of results among NP swabs (CDC-LDT), SN swabs (CDC-LDT), and saliva (Fortitude 2.1), n (%)**		
Saliva only positive	14 (7.5)	20 (13.4)
NP swab only positive	12 (6.4)	4 (2.7)
SN swab only positive	4 (2.1)	9 (6.0)
Saliva and NP swab positive	22 (11.7)	5 (3.4)
NP and SN swab positive	6 (3.2)	3 (2.0)
Saliva and SN swab positive	4 (2.1)	4 (2.7)
All three swabs positive in-tandem	82 (43.6)	15 (10.1)
**Concordance of results among NP swabs (CDC-LDT), SN swabs (Fortitude 2.1), and saliva (Fortitude 2.1), n (%)**		
Saliva only positive	14 (7.5)	23 (15.4)
NP swab only positive	9 (4.8)	5 (3.4)
SN swab only positive	2 (1.1)	4 (2.7)
Saliva and NP swab positive	21 (11.2)	6 (4.0)
NP and SN swab positive	8 (4.3)	2 (1.3)
Saliva and SN swab positive	4 (2.1)	1 (0.7)
All three swabs positive in-tandem	84 (44.7)	14 (9.4)

*NP* nasopharyngeal, *SN* self-administered nasal, *CDC-LDT* Centers for Disease Control and Prevention-Laboratory Developed Test.

**Figure 1:**
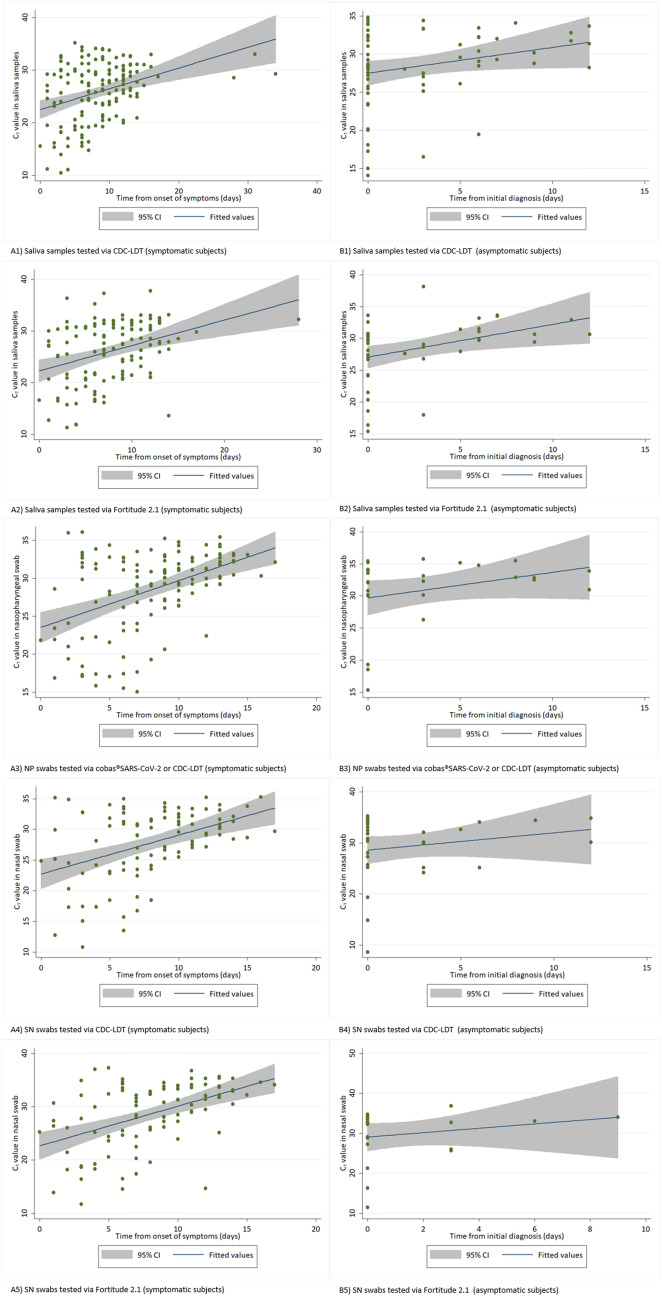
RT-PCR cycle threshold (Ct) values of naso-oropharyngeal saliva samples, nasopharyngeal (NP) swabs, and self-administered nasal (SN) swabs obtained from subjects infected with SARS-CoV-2. (**A**) (left) shows the cycle threshold (Ct) values of samples obtained from symptomatic subjects over time since the onset of symptoms. (**B**) (right) shows the Ct values of samples obtained from asymptomatic subjects over time since the initial COVID-19 diagnosis. Saliva samples and SN swabs were tested via CDC-LDT and Fortitude 2.1. All NP swabs were tested via cobas severe acute respiratory syndrome coronavirus 2 (SARS-CoV-2) (Roche Molecular Systems, Branchburg, NJ) or Centers for Disease Control and Prevention-Laboratory Developed Test (CDC-LDT).

**Figure 2:**
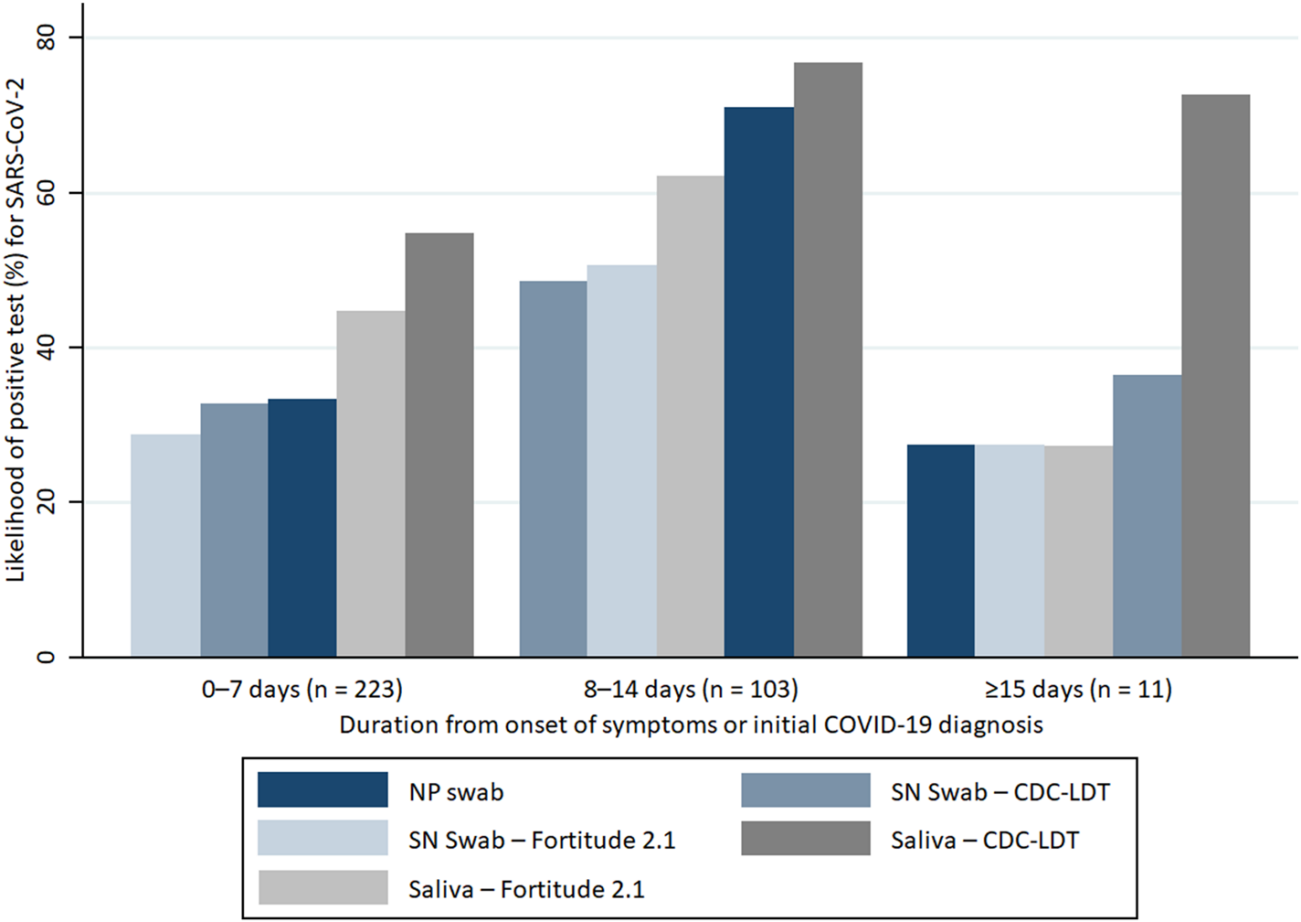
Likelihood of test positivity over time in confirmed COVID-19 subjects. The figure shows the likelihood of positivity for SARS-CoV-2 for nasopharyngeal (NP) swabs, self-administered nasal (SN) swabs, and naso-oropharyngeal saliva samples collected at 0–7 days, 8–14 days, and ≥ 15 days from the onset of symptoms (symptomatic subjects) or initial COVID-19 diagnosis (asymptomatic subjects). NP swabs were tested using via cobas SARS-CoV-2 or Centers for Disease Control and Prevention-Laboratory Developed Test (CDC-LDT).

In total, there were 209 (62.0%) positive saliva tested via CDC-LDT, 167 (49.6%) positive saliva tested via Fortitude 2.1, 150 (44.5%) positive NP swabs tested via cobas SARS-CoV-2 or CDC-LDT, 127 (37.7%) positive SN swabs tested via CDC-LDT, and 119 (35.3%) positive SN swabs tested via Fortitude 2.1. Cycle threshold (Ct) values were lower during the earlier period of infection across all sample types, predominantly for symptomatic infections where the onset of illness could be better estimated (Fig. 1). The likelihood of a positive saliva test was higher than NP and SN from samples collected within weeks 1 and 2 from initial diagnosis. The percentage testing positive for SARS-CoV-2 from any samples fell beyond 14 days of symptom onset in symptomatic subjects or from initial diagnosis for asymptomatic subjects, although this was less significant for saliva tested via CDC-LDT (Fig. 2).

For 63 positive NP swabs with low Ct (< 30) values (the lower results of the two targets), saliva was positive in 62 (98.4%) and 61 (96.8%) samples, while SN swabs were concomitantly positive in 57 (90.5%) and 60 (95.2%) samples when tested via CDC-LDT and Fortitude 2.1, respectively. For 87 positive NP swabs with high Ct (≥ 30) values, saliva was positive in 77 (88.5%) and 64 (73.6%) samples, while SN swabs were concomitantly positive in 49 (56.3%) and 48 (55.2%) samples, respectively. The likelihood of concordant positive tests in other samples was also high when Ct values were low (< 30) in saliva tested via CDC-LDT—76.9% of NP swabs, 64.0% of SN swabs tested via CDC-LDT, and 62.6% of SN swabs tested via Fortitude 2.1 were concomitantly positive. However, only 41.9% of NP swabs, 30.7% of SN swabs tested via CDC-LDT, and 27.4% of SN swabs tested via Fortitude 2.1 were positive in-tandem with saliva samples tested via CDC-LDT when Ct values were high (≥ 30).

Test concordances between different sample sites were relatively good, with the kappa statistic of 0.616 for NP and SN swabs tested via CDC-LDT (agreement of 81.3%), 0.675 for NP tested via CDC-LDT and SN swabs tested via Fortitude 2.1 (agreement of 84.3%), 0.537 for saliva and NP tested via CDC-LDT (76.2%), and 0.602 for saliva tested via Fortitude 2.1 and NP tested via CDC-LDT (80.1%). Test concordances were excellent between the two saliva (87.2%) and SN swabs (91.0%) tests, with a kappa statistic of 0.745 and 0.806, respectively.

**Figure 3:**
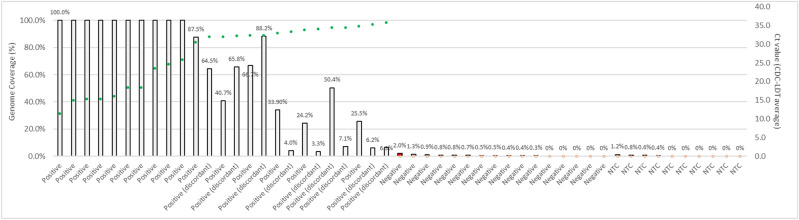
Correlation between viral genome coverage (%) by next generation sequencing (NGS) and cycle threshold (Ct) values for SARS CoV-2. NGS is a sensitive method of detecting low-level SARS-CoV-2 virus in clinical samples, and coverage (%) of the genome is correlated to the Ct value determined by RT-PCR. Thirty samples (17 saliva and 11 SN swabs and 1 NP swab) were tested by NGS. Ten samples (4 saliva, 6 SN swabs) with discordant results from the RT-PCR tests were confirmed to be positive by NGS (median genome coverage 29.7%, range 3.3–88.2%). Genome coverage (%) is defined as the proportion of the SARS-CoV-2 genome that has > 1× depth of coverage. The threshold coverage (%) for making positive call by NGS was established by running 5 negatives (by RT-PCR) samples from this study, 11 negative NP swab samples from community testing, and 10 no-template controls (NTC). The threshold was determined to be the detection of 5 amplicons, which corresponds to a coverage of ~ 1.7% of the SARS-CoV-2 genome. Among five negatives (by both RT-PCR methods) saliva samples, one saliva sample was called positive by NGS (red bar) with a genome coverage of 2.0%. This sample was from a patient with RT-PCR positive NP swab and nasal swab samples collected at the same time, raising the possibility of a low-level signal detected on NGS. Ct values for samples with positive calls (by either or both Fortitude 2.1 and CDC-LDT RT-PCR assays) are represented on the graph with green circles, and samples negative for SARS-CoV-2 (by both RT-PCR tests) and NTC are represented as open circles. Average Ct values (of 2 targets) or single Ct value (when only 1 target was detected) from the CDC-LDT assay are plotted on the secondary axis.

Among 30 samples (saliva, NP and SN swabs) tested by NGS, there was a strong correlation between viral genome coverage by NGS and Ct values for SARS-CoV-2. Ten samples showed 100% coverage (7 unique subjects) (Fig. 3). Ten samples (4 saliva, 6 SN swabs) with discordant results between the 2 RT-PCR tests (CDC-LDT positive, Fortitude negative) were positive by NGS. Phylogenetic analyses of sequences of SARS-CoV-2 viral RNA from high-coverage saliva samples of the 7 unique subjects showed Clade O by GISAID nomenclature^19^ and lineage B.6 by PANGOLIN system of nomenclature^20^.

## Discussion

Our study is concordant with multiple published works supporting saliva as an alternative sample for COVID-19 screening and diagnosis^7–15^, and one of a minority where saliva was shown to be more sensitive than the corresponding NP swab^8,9,13^, although the results by Leung et al. (53.7% saliva vs. 47.4% NP swab, 95 subjects) were not statistically different^8^. Several reasons may account for this difference in the studies, including enrichment from nasal and oropharyngeal secretions, where the viral load is potentially higher^8,9^, or a higher volume of samples collection, where approximately 10 mL of saliva was collected for testing^13^. Steps were taken to minimize biases and errors—NP swabs performed by trained healthcare staff, environmental testing of CAP-accredited laboratory (no evidence of contamination), conduction of tests for most of the samples in the same laboratory, and pre-processing of saliva samples with dithiothreitol before RNA extraction to resolve the issues of saliva specimen viscosity, which can lead to false negatives.

Interestingly but perhaps unsurprisingly, the use of different RT-PCR kits in the present study resulted in different test-positive rates in saliva, suggesting that this can potentially be an important consideration for clinical laboratories, where more sensitive laboratory protocols should be deployed for clinical diagnosis as opposed to mass screening for low-prevalence populations. More validation would be required to confirm this finding.

SN swabs, however, appeared less sensitive compared to both saliva and NP swabs for the diagnosis of COVID-19. Although it was convenient, less time-consuming to perform relative to saliva collection, and caused less discomfort compared to NP swabs, the markedly lower sensitivity should preclude its use where other sample types can be collected.

In our study, NGS provided efficient whole-genome profiling of SARS-CoV-2 for phylogenetic analysis directly from the clinical samples without culture. NGS detection sensitivity was excellent with a threshold of 1.7% genome coverage or 5 amplicons targets, confirming all CDC-LDT positives tested. Other groups have reported highly sensitive performance for NGS with limits of detection ranging between a threshold of 5% genome coverage or 84 genome-equivalents per mL^21^, or at least 5 SARS-CoV-2 targets for detection^22^. The phylogeny results were consistent with the virus belonging to a viral type (Clade O, lineage B.6) known to be circulating in the geographical regions of Singapore and India.

There are several limitations to our work. Firstly, the study population was confined to young and middle-aged men who were either asymptomatic or had mild disease. The results cannot be extrapolated to other populations (e.g., paediatric), where there is a clear need for alternate sample types to NP swabs. Secondly, we did not extend the follow-up testing sufficiently to determine when saliva viral shedding stopped for the majority of subjects, although this has been explored in other studies^7,10^. Thirdly, we did not test for the difference, if any, between saliva obtained from naso-oropharyngeal or the mouth alone, although it is biologically plausible that the latter would result in lower sensitivity for COVID-19 diagnosis^16^.

In conclusion, our study adds to the body of evidence supporting saliva as a sensitive and less intrusive sample for COVID-19 diagnosis and further defines the role of naso-oropharyngeal secretions and the impact of different RT-PCR kits in increasing the sensitivity of testing. In our study, SN swabs were inferior to both saliva and NP swabs. Our study also provides evidence to support NGS in challenging samples for sensitive COVID-19 molecular diagnosis. Such an NGS workflow can also provide direct-from-sample phylogenetic analysis for public health decision-making, such as contact tracing.
